# Assessing the Quality and Clarity of YouTube Videos as an Informational Source for Ankle Fractures: A Study of the Turkish Population

**DOI:** 10.7759/cureus.55891

**Published:** 2024-03-10

**Authors:** Yaşar Samet Gökçeoğlu, Ayşe Nur İncesu, Elif Eda Dağ, Elif Yağmur Özger, Turgut Akgül

**Affiliations:** 1 Orthopedics and Traumatology, School of Medicine, Istanbul University, Istanbul, TUR; 2 Orthopedics and Traumatology, Faculty of Medicine, Istanbul University, Istanbul, TUR; 3 Orthopedics and Traumatology, Faculty of Nursing, Istanbul University, Istanbul, TUR; 4 Physical Medicine and Rehabilitation, Istanbul University Cerrahpaşa School of Medicine, Istanbul, TUR

**Keywords:** ankle fracture, video sharing, information quality, information literacy, health education, informatics, medicine

## Abstract

Background and objective

The significance of YouTube as a prominent source of healthcare-related information cannot be overstated. Its influence on patient education is substantial. However, despite its popularity, there has been little research into the quality and comprehensibility of videos related to ankle fractures, a condition with increasing incidence rates, particularly in the context of Turkey. To address this gap in the literature and the growing reliance on digital platforms for health information during the coronavirus disease 2019 (COVID-19) pandemic, this study aims to assess the quality, clarity, and pedagogical value of YouTube videos on ankle fractures for the Turkish population.

Methodology

A comprehensive examination of 150 YouTube videos concerning ankle fractures, employing exacting inclusion and exclusion criteria to identify 52 videos for in-depth analysis was undertaken. The investigation entailed an assessment of content quality, presentation style, and user interaction, utilizing the criteria established by the Journal of the American Medical Association (JAMA) and the Patient Education Materials Assessment Tool (PEMAT), as well as a novel measurement known as the Ankle Fracture Content Score (AFCS). A statistical analysis was executed to gauge the educational value and dependability of the information presented.

Results

The majority of ankle fracture management videos were found to be uploaded by medical professionals, covering various aspects such as rehabilitation and patient testimonials. However, the overall educational quality was suboptimal, with patient-shared videos lacking in depth and accuracy. Statistical analysis showed no significant differences in content quality across different uploader categories, except for notably lower educational quality in videos shared by patients.

Conclusions

The findings underscore a significant need for healthcare professionals and academic institutions in Turkey to produce and share high-quality, reliable, and comprehensible videos on ankle fractures. Leveraging YouTube's extensive reach can significantly improve health literacy among the Turkish public, ensuring access to accurate and trustworthy health information.

## Introduction

YouTube serves as a vast repository of healthcare-related videos, featuring content contributed by a diverse array of users, including medical practitioners, patients, and manufacturers of biomedical devices [1]. Previous research has indicated that a substantial proportion of patients, approximately 75%, rely on online sources to make healthcare decisions. Moreover, nearly 7% of the daily eight billion Google searches are health-related [2]. Given its close affiliation with Google, YouTube attracts a remarkable number of daily views and boasts a substantial user base of over 2 billion active users per month [3]. Additionally, it is increasingly being utilized as a means to disseminate health information [4].

While recent studies on healthcare-related YouTube videos have revealed that they generally lack the ability to accurately depict medical diagnoses and treatments for common illnesses and procedures, limited research has explored the ease with which patients can comprehend and absorb this information when watching these videos [5]. The increasing trend of using YouTube for healthcare information, coupled with the rise in unreliable and misleading videos, can lead to confusion and frustration among both healthcare providers and patients [6].

It is crucial for physicians to understand the quality of information available to patients regarding ankle fractures when discussing diagnoses and treatment options, as quality patient education has been proven to decrease anxiety and improve outcomes. Although some studies have assessed educational content on YouTube for a variety of orthopedic injuries and conditions, no studies have specifically examined content related to ankle fractures [7]. Given the significant rise in ankle fracture cases, reported at 419.9 per 100,000 in a comprehensive study covering 204 countries in 2019 an increase of 33.4% since 1990-and the crucial role of YouTube as an informational resource during periods of extended home confinement, such as the coronavirus disease 2019 (COVID-19) pandemic, the importance of digital platforms in public health education has been emphasized [8]. The recent surge in the literature concerning YouTube, particularly in the context of the United States, where daily search queries related to ankle injuries exceed 7,000, exemplifies this trend. At the same time, the COVID-19 pandemic has led to an increased reliance on the internet in our society for health-related inquiries, with platforms like YouTube becoming a primary resource for individuals seeking information about their medical conditions. However, there is a noticeable gap in the literature regarding the Turkish population's ankle fractures. This study aims to evaluate the quality, clarity, and pedagogical value of YouTube videos as an informational source in this context, considering the observed rise in ankle fracture incidences and the growing engagement with internet resources among the general public.

## Materials and methods

Study design

A comprehensive investigation of the content on YouTube related to "ankle fractures" was undertaken to assess its availability and quality. Google Chrome (version 121.0.6167.139, Official Build, arm64) was utilized in private browsing mode with all cookies cleared prior to the search and a freshly created YouTube account was employed to ensure an unbiased collection of data. Searches were conducted using the initial 150 videos listed in the search results for "ankle fracture," a number informed by previous research indicating that YouTube users typically do not navigate beyond the initial set of search results.

To guarantee the reliability and consistency of the selected videos, two independent reviewers, trained on a predetermined set of criteria, will evaluate the videos for content quality, informational value, presentation style, and user interactions. These criteria are essential in assessing the educational value and pertinence of the videos to individuals seeking information on ankle fractures. The analysis of the videos was carried out on February 18, 2024, to ensure the timeliness and relevance of the study's findings to current online content trends. This robust methodology serves to reveal the quality of information accessible to the public on YouTube regarding ankle fractures, highlighting areas of excellence and opportunities for improvement.

Data collection and video evaluation

In this research endeavor, we conducted a systematic collection of the initial 150 videos from YouTube that met our predetermined search criteria for "ankle fractures." Our selection process included specific exclusion criteria to streamline the pool of videos for analysis. We disregarded videos without audio, those with a duration of less than 90 seconds, those unrelated to ankle fractures, those in a non-Turkish language, and duplicate content. Ultimately, 52 videos were deemed appropriate for further analysis.

For each video selected for the study, we diligently recorded various characteristics to enable a thorough evaluation. These characteristics comprised the video's title, duration, number of views, uploader/source, comment count, age (in days since upload), view rate (calculated as views per day), likes, dislikes, like/dislike ratio (expressed as a percentage), Video Power Index (VPI), subscriber count, and YouTube verification status [4,5]. We categorized video sources into five distinct categories: academic sources (affiliated with educational institutions or research programs), physicians, non-physician healthcare professionals (such as physical therapists or physician assistants), patients, and device companies.

To assess the credibility and dependability of the information, we utilized the criteria set forth by the Journal of the American Medical Association (JAMA). This assessment method entails four distinct criteria, each assigned a score of 1, to offer a general evaluation of source reliability. A maximum score of 4 signifies exceptional accuracy and reliability, while a score of 0 indicates poor accuracy and reliability. Although the scoring system has not been formally validated, it has been widely employed in prior research to assess the trustworthiness of online information sources [9,10].

Additionally, we applied two separate scoring systems to gauge the educational value of the videos on ankle fractures. The Patient Education Materials Assessment Tool (PEMAT) was utilized as a validated instrument to measure the comprehensibility and practicality of the content (tables present in the Appendices). The comprehensibility was evaluated through 21 specific criteria, while the practicality was assessed using four criteria [11]. We calculated total scores for comprehensibility and practicality, which were then converted into percentages by dividing them by the corresponding number of items. A higher percentage indicated a video with superior comprehensibility or practicality, thus providing a more accurate measure of its educational value [12].

The Ankle Fracture Content Score (AFCS) presents a novel approach to evaluating online video material, particularly emphasizing their educational value concerning ankle fractures for both patients and medical providers. Recognizing that videos intended for healthcare professionals often appear in patient searches, this scoring system assesses the depth and breadth of content provided universally [9,13].

The AFCS system was developed in collaboration with two orthopedic trauma surgeons who possess fellowship training and active practice experience. The AFCS has earned an official endorsement for its application. The AFCS is built upon a foundation of patient-oriented information from reputable sources such as the Orthopaedic Trauma Association (OTA), the American Academy of Orthopaedic Surgeons (AAOS), the American Podiatric Medical Association (APMA), the American Orthopaedic Foot & Ankle Society (AOFAS), and the American College of Foot and Ankle Surgeons (ACFAS). The comprehensive nature of this source material ensures the scoring system is both comprehensive and reflective of current standards in patient education regarding ankle fractures [9,14].

The scoring methodology of the AFCS is divided into two primary categories: major and minor criteria. Major criteria are considered essential elements for foundational patient education on ankle fractures, and they are assigned a value of two points each. Minor criteria, while still beneficial for patient education, are considered less critical for a fundamental understanding of the condition and are assigned one point each. This bifurcated system allows for a maximum achievable score of 14 points, with higher scores indicating videos of superior educational quality.

The introduction of the AFCS scoring system aligns with ongoing efforts to refine and systematize the evaluation of medical information online. This framework offers researchers and authors a structured approach for assessing the educational merit of videos on specific medical topics.

Statistical analysis

To ensure the consistency and reliability of our evaluation, we employed the intraclass correlation coefficient (ICC), a statistical measure effective in quantifying the level of agreement among ratings. This method was used to assess inter-rater reliability and provide a robust assessment of our evaluation's precision. Initial data analysis involved applying the Shapiro-Wilk test, a crucial procedure aimed at evaluating the normality of the data distribution. This preliminary analysis determined the appropriate statistical methods for subsequent comparative evaluations. For data exhibiting a normal distribution, analysis of variance (ANOVA) was used. This statistical approach facilitated the comparison of means across multiple groups, suitable for normally distributed datasets. Conversely, for data that did not follow a normal distribution, the Kruskal-Wallis test was utilized. This non-parametric method is particularly adept at comparing median values across different groups without the assumption of normality. For the examination of relationships involving dual numerical scales, Pearson’s Correlation testing remained a critical tool. It allowed for the exploration of linear associations between variables, offering insights into the strength and direction of these relationships. All statistical analyses were conducted using IBM SPSS Statistics for Windows, Version 28 (Released 2021; IBM Corp., Armonk, New York, United States). A P-value of less than 0.05 was considered statistically significant.

## Results

**Figure 1:**
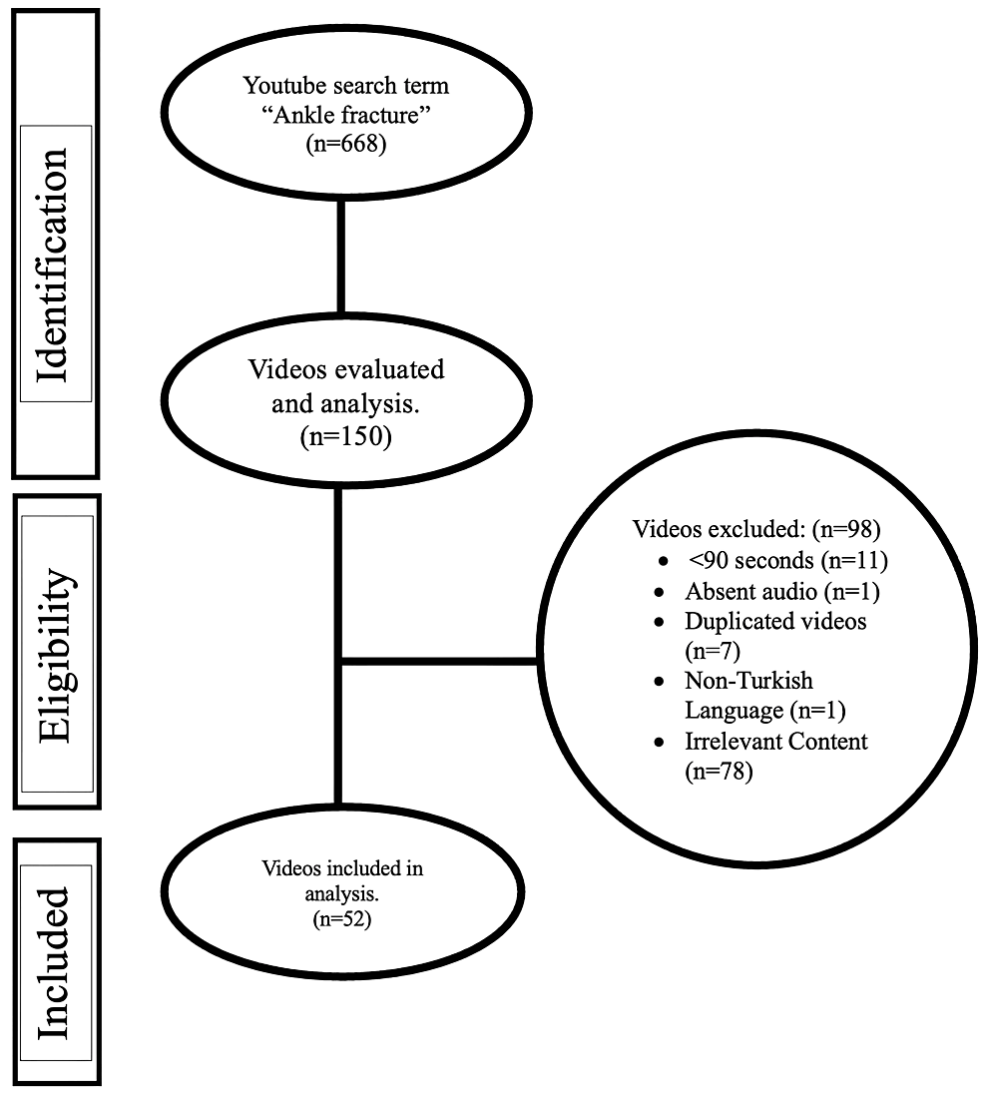
Evaluation strategy employed for identifying YouTube videos related to ankle fractures.

An analysis was conducted on 52 YouTube videos related to ankle fractures, selected from an initial pool of 150 videos. These videos were subjected to a detailed evaluation process to ensure they met the established inclusion criteria. The exclusion of videos was based on several factors, including duration of less than 90 seconds, irrelevant content, non-Turkish language, absence of audio, and duplicate content (Figure 1). The videos were uploaded between June 2014 and January 2024, with the majority originating from medical professionals (n=28, 53.84%), followed by academic entities (n=2, 3.84%), non-physician health practitioners (n=6, 11.53%), commercial sources such as device companies (n=3, 5.76%), and patients (n=13, 24.99%).

The average length of the videos was 5.05 minutes, with a standard deviation of 7.22 minutes, and a range from 1.5 to 49.05 minutes. The combined metrics for views, likes, and comments were 2,367,935, 17,673, and 10,438, respectively, yielding an average daily view rate of 35.41 ± 45.33. Comments were disabled for four videos, and none of the videos featured an active dislike option. The mean period since the video upload was recorded at 1354.15 days, with a standard deviation of 1466.04 days, and a range from 24 to 9490 days (Tables 1, 2).

**Table 1:** Youtube video features. Min: Minimum; Max: Maximum

Characteristic	Average	Standard deviation	Min	Max
Video duration, minutes	5.05	7.22	1.5	49.05
Days since upload, days	1354.15	1466.04	24	9490
Views	45537.21	67707.27	11	309791
Likes	339.86	690.07	0	4200
View ratio	35.41	45.33	0.03	257.4
Comments	217.45	449.21	0	2894
Source subscriber number	10288.36	18567.03	11	108000
Video power index (VPI)	4910.67	8483.37	0	35040

**Table 2:** Youtube video characteristics.

Characteristic	Number of videos (%)
Youtube source	
Academic	2 (3.84%)
Physician (Doctor)	28 (53.84%)
Physiotherapist	6 (11.53%)
Patient	13 (24.99%)
Device company	3 (5.76%)
Youtube verification presence	0
Medical disclaimer provided	0

Eleven videos were produced to focus on the rehabilitation and physical therapy aspects of managing ankle fractures, providing guidance on exercises and stretches. Out of these, 13 videos featured patient testimonials (n=13, constituting 24.99% of the total). A substantial portion of the videos included medical disclaimers (n=28, amounting to 53.84%). The JAMA criteria received a good consensus from observers, while the AFCS and the PEMAT rated the content as excellent. The average scores assigned by evaluators according to these assessment systems, along with the ICC values for interobserver agreement, are presented in Table 3.

**Table 3:** Interobserver agreement assessments based on scoring systems. JAMA: Journal of the American Medical Association; PEMAT: Patient Education Materials Assessment Tool; AFCS: Ankle fracture content score; Min: Minimum; Max: Maximum; ICC: Intraclass correlation coefficient; SD: Standard deviation; CI: Confidence interval

Criteria	Observer	Average	SD	Min.	Max.	ICC (95% CI)
JAMA	1	1.78	0.61	0	4	0.754 (0.606-0.852)
	2	2.52	0.97	1	4	
PEMAT – Understandability	1	7.21	2.16	1	5	0.942 (0.901-0.966)
	2	6.95	2.04	1	5	
PEMAT – Actionability	1	1.9	0.6	18	60	0.984 (0.972-0.991)
	2	1.8	0.7	17	62	
AFCS	1	5.55	3.08	1	13	0.962 (0.934-0.978)
	2	5.85	3.12	1	13	

In a comparative examination of video material from YouTube concerning "ankle fractures," our statistical evaluation did not reveal any substantial differences based on the JAMA and PEMAT scores, with P-values of 0.12 and 0.06, respectively. This suggests that there is uniformity in content quality across various uploader categories. However, an exception was observed in the AFCS, where videos shared by patients were found to significantly lag in educational quality in comparison to those from other sources, as evidenced by a significantly lower AFCS (P<0.001). The videos lacked YouTube verification and medical disclaimers, indicating a gap in content credibility and accountability. The uploader demographic was diverse, with an average subscriber count of 10,288 ± 18,567.03, highlighting content creators ranging from individuals to large channels. The JAMA score averaged at 1.98 ± 0.61, mainly because of the prevalent absence of references in the videos, which mostly scored 2 points. The PEMAT assessment aimed to understand and actionability, yielded average scores of 7.21 ± 2.16 and 1.9 ± 0.60, respectively, with no significant correlation found with other variables (P>0.05). This extensive analysis highlights the variability in educational content on ankle fractures available on YouTube, particularly emphasizing the shortcomings in patient-shared videos in terms of depth and accuracy as per the AFCS evaluation.

## Discussion

The potential of YouTube as a platform for disseminating patient information is undeniable. However, its open publication model also poses the risk of disseminating misleading or incorrect information, which could jeopardize patient care. Our analysis lends support to the notion that the quality of content related to "ankle fractures" on YouTube is suboptimal. To the best of our knowledge, no other study within the Turkish population has explored the educational content on ankle fractures available on YouTube or other platforms. Previous analyses on YouTube regarding various pathologies have investigated the reliability and educational value of existing videos, with similar outcomes. However, few have assessed the understandability and applicability of the information provided through a validated system like PEMAT.

This study aligns with the broader findings that the overall educational value of videos on YouTube remains weak. However, it delves deeper by considering the role of the target audience. It also discusses YouTube's recent initiatives to prioritize health content shelves. By highlighting the essential factors needed while providing high-quality information, this research aims to further raise awareness of the educational gaps in current patient education [3-5].

Our research also revealed that PEMAT and JAMA scores were similar across patient populations and videos published by doctors, healthcare professionals, and university publications. This similarity suggests that doctors or healthcare workers may publish videos carelessly to increase their popularity. Furthermore, the study highlights a significant scarcity of educational videos on YouTube, a major social media platform where patients frequently seek information about their conditions. Our findings underscore the need for enhanced dissemination of accurate information on social media platforms and recommend that associations or university entities address their shortcomings in social media engagement to improve public knowledge.

One aspect of our study that merits consideration is the potential for selection bias resulting from our reliance on specific keywords and a single language for conducting YouTube video searches. By doing so, we may inadvertently overlook a broader range of educational content that could be relevant to our inquiry. Furthermore, the dynamic nature of YouTube, characterized by the continuous uploading and removal of videos, could impact the representativeness of our sample over time. This, in turn, may limit the generalizability of our findings to a wider audience or different time frames.

In the existing literature, a similar study conducted in the United States searched for "ankle fractures" on YouTube and analyzed only the first 100 videos, yielding a sample of 62 videos. The findings of this earlier study align with our own, as both studies reveal low scores across AFCS, JAMA, and PEMAT, indicating a general trend of suboptimal educational quality in YouTube videos about ankle fractures. This parallel observation suggests a more widespread issue affecting the educational content available on YouTube for medical topics, transcending geographical boundaries [4].

Comparing our study's results with those of other YouTube studies in different medical fields reveals a consistent pattern: the educational quality of health-related videos varies greatly, with many falling short of established quality and reliability standards. Research in areas such as rehabilitation, hemophilia, and overactive bladder has also identified disparities in video content quality. These findings underscore the need for healthcare professionals to guide patients toward credible information sources, given the ubiquity of this problem across a wide range of medical topics. Comparing our study with others highlights a global challenge in health education on digital platforms and underscores the importance of improving the accuracy and reliability of online health information [15-17].

## Conclusions

Our research unveils a striking inadequacy of exceptional educational content on ankle fractures specifically designed for the Turkish population on YouTube. This highlights the essential need for healthcare professionals and academic institutions in Turkey to actively engage in creating and disseminating precise, dependable, and understandable informational resources. By doing so, they can leverage YouTube's extensive reach to guarantee accessible and trustworthy health information, thereby promoting health literacy among the Turkish public.

## Appendices

 The PEMAT is a validated tool that measures the understandability and actionability of information available to patients.  Understandability was measured using 14 criteria, while actionability was measured using seven criteria. Total scores for understandability and actionability were calculated and divided by the number of items for each, which yielded a percentage. A higher percentage indicated a video that was more understandable or actionable [4,11].

**Table 4:** Understandability Criteria for Patient Education Materials Assessment Tool (PEMAT) Audio/Visual Materials. Source: [4,11]

Understandability			
Item	Item	Response Options	Rating
Topic: Content			
1	The material makes its purpose completely evident.	Disagree=0, Agree=1	
Topic: Word Choice & Style			
2	The material uses common, everyday language.	Disagree=0, Agree=1	
3	Medical terms are used only to familiarize audience with the terms. When used, medical terms are defined.	Disagree=0, Agree=1	
4	The material uses the active voice.	Disagree=0, Agree=1	
Topic: Organization			
5	The material breaks or "chunks" information into short sections.	Disagree=0, Agree=1, Very short material^i^=N/A	
6	The material's sections have informative headers.	Disagree=0, Agree=1, Very short material^i^=N/A	
7	The material presents information in a logical sequence.	Disagree=0, Agree=1	
8	The material provides a summary.	Disagree=0, Agree=1, Very short material^i^=N/A	
Topic: Layout & Design			
9	The material uses visual cues (e.g., arrows, boxes, bullets, bold, larger font, highlighting) to draw attention to key points.	Disagree=0, Agree=1, Video=N/A	
Topic: Use of Visual Aids			
10	The material uses visual aids whenever they could make content more easily understood (e.g., illustration of healthy portion size).	Disagree=0, Agree=1	
11	The material’s visual aids reinforce rather than distract from the content.	Disagree=0, Agree=1, No visual aids=N/A	
12	The material’s visual aids have clear titles or captions.	Disagree=0, Agree=1, No visual aids=N/A	
13	The material uses illustrations and photographs that are clear and uncluttered.	Disagree=0, Agree=1, No visual aids=N/A	
14	The material uses simple tables with short and clear row and column headings.	Disagree=0, Agree=1, No tables=N/A	

**Table 5:** Actionability Criteria for Patient Education Materials Assessment Tool (PEMAT) Audio/Visual Materials. Source: [4,11]

Actionability			
Item	Item	Response Options	Rating
1	The material clearly identifies at least one action the user can take.	Disagree=0, Agree=1	
2	The material addresses the user directly when describing actions.	Disagree=0, Agree=1	
3	The material breaks down any action into manageable, explicit steps.	Disagree=0, Agree=1	
4	The material provides a tangible tool (e.g., menu planners, checklists) whenever it could help the user take action.	Disagree=0, Agree=1	
5	The material provides simple instructions or examples of how to perform calculations.	Disagree=0, Agree=1, No calculations=NA	
6	The material explains how to use the charts, graphs, tables, or diagrams to take actions.	Disagree=0, Agree=1, No charts, graphs, tables, or diagrams=N/A	
7	The material uses visual aids whenever they could make it easier to act on the instructions.	Disagree=0, Agree=1	
